# Magnesium alloy covered stent for treatment of a lateral aneurysm model in rabbit common carotid artery: An in vivo study

**DOI:** 10.1038/srep37401

**Published:** 2016-11-21

**Authors:** Wu Wang, Yong-Li Wang, Mo Chen, Liang Chen, Jian Zhang, Yong-Dong Li, Ming-Hua Li, Guang-Yin Yuan

**Affiliations:** 1 Institute of Diagnostic and Interventional Radiology, Shanghai Jiao Tong University Affiliated Sixth People’s Hospital, Shanghai, 200233, China; 2 Department of Interventional Radiology, Shanghai Jiao Tong University Affiliated Sixth People’s Hospital, (Fengxian Hospital), Shanghai, China; 3 National Engineering Research Center of Light Alloy Net Forming and State Key Laboratory of Metal Matrix Composite, Shanghai Jiao Tong University, Shanghai 200240, China

## Abstract

Magnesium alloy covered stents have rarely been used in the common carotid artery (CCA). We evaluated the long-term efficacy of magnesium alloy covered stents in a lateral aneurysm model in rabbit CCA. Magnesium alloy covered stents (group A, n = 7) or Willis covered stents (group B, n = 5) were inserted in 12 New Zealand White rabbits and they were followed up for 12 months. The long-term feasibility for aneurysm occlusion was studied through angiograms; the changes in vessel area and lumen area were assessed with IVUS. Complete aneurysmal occlusion was achieved in all aneurysms. Angiography showed that the diameter of the stented CCA in group A at 6 and 12 months was significantly greater than the diameter immediately after stent placement. On intravascular ultrasound (IVUS) examination, the mean lumen area of the stented CCA in group A was significantly greater at 6 and 12 months than that immediately after stent placement; the mean lumen area was also significantly greater in group A than in group B at the same time points. The magnesium alloy covered stents proved to be an effective approach for occlusion of lateral aneurysm in the rabbit CCA; it provides distinct advantages that are comparable to that obtained with the Willis covered stent.

Magnesium alloys have low density, high intensity, good biocompatibility, and anti–platelet aggregation properties, and also have the advantage of degrading *in vivo*^1^. Furthermore, the degradation products are easily removed from the body^2^. Although, magnesium alloy stents have been used in European hospitals^3^,^4^, some important questions still need to be answered, for example, regarding their degradation *in vivo* and the changes induced in the stented vessel.

Recently, our collaborators have successfully developed a quaternary magnesium alloy (Mg-Nd-Zn-Zr) and manufactured microtubes with low dimensional error for stent fabrication^5^,^6^,^7^,^8^. The magnesium alloy covered stent used in this study was jointly developed by MicroPort Medical Company (Shanghai, China) and Shanghai Jiao Tong University. In this study, we used the covered stent for occlusion of a lateral aneurysm model in rabbit common carotid artery, which has not been reported in the literature. The purpose of this study was to evaluate the long-term efficacy of the magnesium alloy covered stents for treatment of lateral aneurysm in the rabbit CCA, its *in vivo* degradation behavior, and the intravascular ultrasound grayscale results.

## Materials and Methods

### Stent characteristics

**Figure 1:**
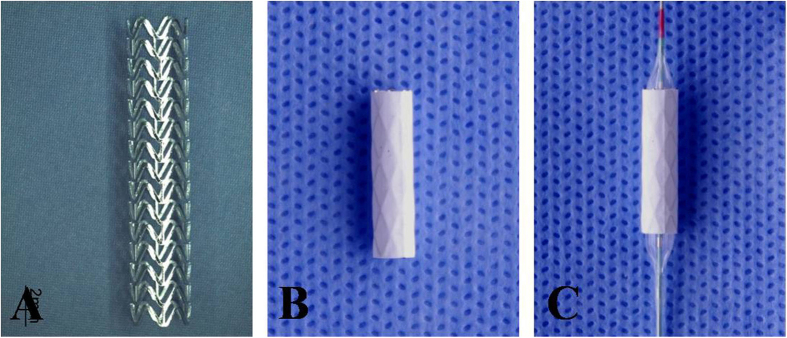
The magnesium alloy covered stent. (**A**) The magnesium alloy bared stent; (**B**) the e-PTFE membrane is glued along the length of the stent struts; (**C**) the magnesium alloy covered stent mounted on a balloon catheter with an outside diameter of 5 F.

The magnesium alloy covered stents consists of three parts: a magnesium alloy bared stent, an expandable polytetrafluoroethylene (e-PTFE) membrane, and a balloon catheter (Fig. 1). The bared stent is sculpted by a laser from a magnesium microtube. Table 1 shows the composition of the magnesium alloy. The procedure for processing of the magnesium alloy microtubes has been described by us before^5^,^6^,^7^,^9^. Briefly, the magnesium alloy ingot was prepared by semi-continuous casting. After extrusion at 350 °C (extrusion ratio 20), a Φ22 mm rod was prepared. Magnesium alloy microtubes were manufactured following extrusion, drawing, and annealing, and the stents were crafted out of the microtubes by pulsed laser cutting, followed by annealing, acid washing, and electropolishing. The final stent size was Φ3 × 15 mm, with a wall thickness of 150 μm. The e-PTFE, in a tubular configuration with a thickness of 30–50 μm, is glued along the length of the stent struts. The balloon catheter (MicroPort Medical Company, Shanghai, China) is a rapid-exchange system, with a length of 145 cm. The stent is premounted on the balloon catheter, which has an outside diameter of 5 F.

The Willis covered stent also consists of three parts (Fig. 1)^10^,^11^,^12^: a bared stent, an e-PTFE membrane, and a balloon catheter. The bared stent, with a segmented design and open cell structure, is laser-sculpted from a cobalt-chromium superalloy microtube (60 μm in diameter). The e-PTFE membrane and the balloon catheter used with the Willis stent were as described before. The Willis covered stent was also premounted on a balloon catheter with an outside diameter of 3.8 F (1.27 mm).

The magnesium alloy covered stents (group A, n = 7) and the Willis covered stents (group B, n = 5) were implanted into the CCAs of New Zealand white rabbits.

### Animals

All experimental procedures were performed in accordance with the National Institutes of Health guidelines for humane handling of animals and were approved by the animal research committee of Shanghai Jiao Tong University Affiliated Sixth People’s Hospital. Twelve New Zealand rabbits of both genders, weighing 2–2.5 kg, were studied between January 2013 and July 2015. All animals were maintained on a standard laboratory diet.

### Establishment of the common carotid artery lateral aneurysm model

One week after admission of the rabbits to our laboratory, the left CCA of all rabbits were ligated, the aim being to thicken the right CCA to facilitate aneurysm making.

The CCA lateral aneurysm model was established 1 month later. After anesthesia with intravenous injection of 1 mL/kg pentobarbital, the rabbit was fixed on the table in a supine position. Under sterile conditions, an approximately 4-cm long horizontal skin incision was made below the central thyroid cartilage, deviating to the right. The hypodermis was bluntly dissected and a 2-cm segment of the right external jugular vein was exposed. The vein was ligated at the distal and proximal ends with a no. 4 suture, and a 1-cm long venous pouch without branches, with one end ligated and the other open, was excised. The pouch was rinsed with heparinized saline solution delivered through a smooth needle syringe, and trimmed. All pouches were stored in heparinized saline solution till use. Next, the anterior cervical muscle group on the right (i.e., the sternocleidomastoid and sternohyoid muscles) was bluntly dissected to reveal the right common carotid artery. One centimeter of the right common carotid artery was isolated and temporarily clipped proximally and distally with two atraumatic hemostatic clamps. After stripping the adventitia, an incision was made between the two clamps and the duct cavity was rinsed with heparinized saline solution. An end-to-side anastomosis was then created between the free end of the venous pouch and the CCA wall using discontinuous extroversion sutures with 6–0 stitches. After the anastomosis was created, the clamps were loosened and the venous pouch was allowed to fill completely. The aneurysm neck was checked for bleeding, and a tight suture was placed if necessary.

After surgery, the muscle and skin were layer-sutured. Penicillin (400,000 U/d) and low molecular weight heparin calcium (100 U/kg/12 h) were administered for 3 days postoperatively.

### Covered stent placement

All procedures were performed by two of the authors (Y. D. L. and W.W.). After anesthesia, the right femoral artery was isolated, and a 6 F sheath was introduced. The procedure of covered stent placement has been previously described^10^,^11^,^12^.

All animals received postoperative antibiotic (penicillin 400,000 U/d) for 3 days. Low molecular weight heparin calcium (100 U/kg/12 h) was also administered for 3 days postoperatively, after which the animals received aspirin and ticlopidine orally for 6 months to prevent thrombosis and in stent stenosis.

### Degradation behavior of the magnesium alloy stent

#### Magnetic resonance angiography examination

Magnetic resonance angiography (MRA) examination was performed at 1, 2, 4, 5, and 6 weeks after stent placement. All MRA examinations were performed using a 3.0-T system (Achieva X-Series, Philips Medical Systems) with a Sense-Head-8 receiver head coil. The details of the MRA image acquisition and postprocessing approaches have been previously described^13^,^14^.

#### Molybdenum target examination

Molybdenum target examination was performed at 2, 4, 6, 9, and 12 months after stent placement. After anesthesia, the rabbit was fixed in a prone position, and posteroanterior and oblique projections of the neck were obtained on a digital molybdenum target X-ray monoplanar unit (Siemens Mammomat, Inspiration 3117, Siemens) with the following parameters: 56–60 kVp, 28–32 mA, and acquisition time of 3 seconds.

### Postoperative outcome evaluation

#### Follow-up angiographic evaluation

The follow-up angiographic examinations were performed at 3, 6, and 12 months after the stent placement. All the angiographic follow-up images were obtained in posteroanterior and lateral projections along with a calibrated ruler to measure the diameter of the stent and the stenosis of the stented CCA. Digital measurements were made using Photoshop 8.0 (Adobe Systems, Palo Alto, CA, USA), and the average of multiple measurements at the three parts of the stented CCA (proximal, middle, and distal) were used for the analysis. Analyses of the angiographic findings were based on the consensus of three observers.

### IVUS grayscale analysis

We used the IVUS (ILAB^TM^ Ultrasound Imaging System, H749ILAB220CART0, Boston Scientific, Natick, MA, USA) with a 40-MHz catheter (Atlantis, SR Pro, Boston Scientific, Natick, MA, USA). The catheter was placed 10 mm distal to the stent and a complete imaging run was performed using 0.5 mm/s automated pullback to a position 10 mm proximal to the treated segment. These IVUS examinations were recorded on digital versatile discs for quantitative analysis. The vessel area, scaffold area, lumen area, intrascaffold neointimal area, and luminal area stenosis were measured with a computer-based contour detection program. The percentage of lumen area stenosis was calculated as [(the mean lumen scaffold area − the lumen area within the scaffold) ÷ mean lumen scaffold area] × 100^15^.

### Statistical analysis

All the data were expressed as means ± standard deviation. Differences between the two treatment groups were assessed using the nonparametric chi square test or Fisher’s exact test. Comparisons of the variables between the two groups were performed by applying the Mann–Whitney test for paired values. Statistical analysis was performed using SPSS for Windows version 13.0 (SPSS Inc., Chicago, IL, USA). *P* ≤ 0.05 was considered statistically significant.

## Results

### Aneurysm creation, stent placement, and follow-up

The right CCA lateral aneurysm model was successfully established in all twelve rabbits. Digital subtraction angiography (DSA) before stent placement demonstrated the lateral aneurysm in all twelve rabbits, with small amounts of thrombosis in four aneurysms. Six of the aneurysms were located at the middle portion of the right CCA, two in the upper portion, and four in the lower portion.

**Figure 2:**
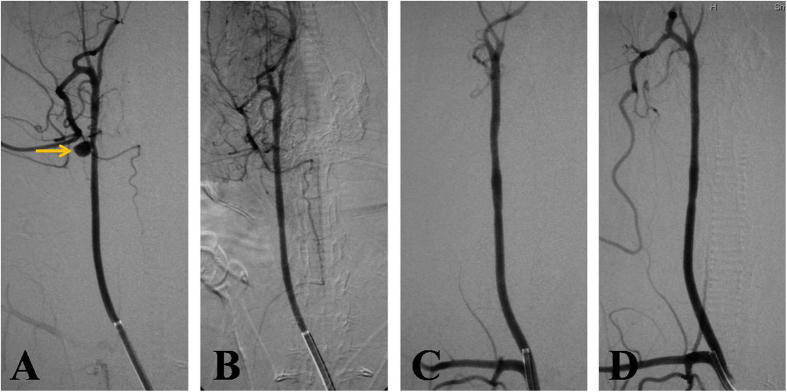
The magnesium alloy covered stent implantation and follow-up angiography of a rabbit lateral aneurysm model. (**A**) Preoperative DSA shows a lateral aneurysm at the upper portion of the CCA; (**B**) DSA immediately after stent placement demonstrates the disappearance of the aneurysm; (**C**,**D**) DSA 6 and 12 months after stent placement displays the disappearance of the aneurysm, without obvious stenosis of the stented CCA.

Stent placement was technically successful in all rabbits without any procedure-related complications. Complete aneurysmal occlusion without endoleak was achieved in eleven aneurysms (Fig. 2), and transient endoleaks into the aneurysm sac were observed in one aneurysm immediately after initial covered stent deployment. Stenosis (>30%) of the stented common carotid artery was observed in one rabbit with a magnesium alloy covered stent immediately after stent placement; this resolved 3 months after stent placement.

All rabbits were alive at the end of the 6 or 12 months of follow-up.

### Angiographic results

The angiographic findings after stent placement are summarized in Table 2. In group A, the diameter of the stented CCA at 6 and 12 months was significantly greater than the diameter immediately after stent placement (*P* < 0.05). There was no significant difference between the diameters at 6 and 12 month (*P* > 0.05). Compared with the diameter immediately after stent placement, the average diameter of the stented CCAs at 6 and 12 months increased by 10.97% and 11.93%, respectively.

In group B, the diameter of the stented CCAs at 6 and 12 months was significantly smaller than the diameter immediately after stent placement. There was no significant difference between the diameters at 6 and 12 months (*P* > 0.05). Compared with the diameter immediately after stent placement, the average diameter of the stented CCAs at 6 and 12 months decreased by 5.37% and 7.85%, respectively.

In addition, there were no significant differences between the groups in the diameters of the same stented sections immediately after stent placement, but the diameters of group A were significantly bigger than those of group B at the 6 and 12 months follow-ups (P < 0.05; Table 2).

### Degradation of the magnesium alloy stent

**Figure 3:**
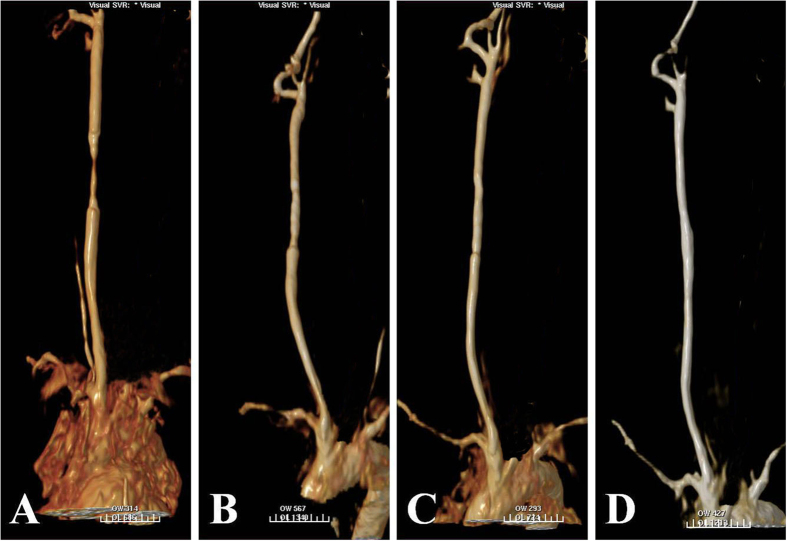
MRA demonstrates the degradation of the magnesium alloy stent with time. (**A**) The morphology of the stented CCA presented single loss on MRA with an appearance similar to severe stenosis, on day 1 after stent placement due to the existence of Nd. (**B**,**C**) The morphology of the stented CCA gradually became normal 2–3 weeks after stent placement with the degradation of Nd. (**D**) The morphology of the stented CCA became normal at 4–5 weeks after stent placement with the degradation of Nd on the surface of the stent.

Figure 3 shows the morphological changes in the stented CCAs as revealed by MRA. The morphology of the stented CCA presented single loss similar to severe stenosis on day 1 after stent placement due to the presence of Nd, but the morphology gradually became normal 5–6 weeks after stent placement with the degradation of Nd.

**Figure 4:**
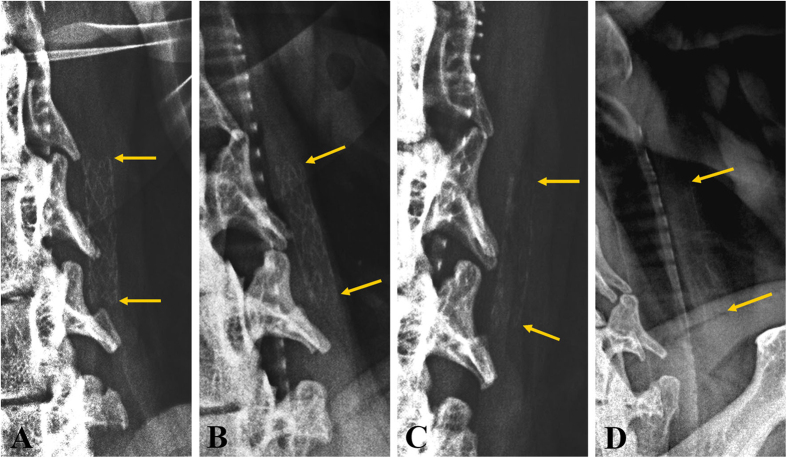
Degradation of the magnesium alloy stent with time on molybdenum target examination. (**A**) Molybdenum target shows the the magnesium alloy stent in the right CCA, with integration of the stent structure and clear outlines 2 weeks after stent placement. (**B**,**C**) The magnesium alloy stent gradually disintegrated, and the contour of the stent became vague under molybdenum target at 2 and 6 months follow-up. (**D**) The contour of the stent could not be easily visualized under molybdenum target at 1 year follow-up.

Figure 4 shows the degradation of the magnesium alloy stent with time on molybdenum target examination. The morphology of the magnesium alloy stent was intact and clearly visualized for up to 2 weeks after stent placement. The stent then gradually disintegrated, with the contour becoming progressively vague, until it could not be easily visualized under molybdenum target at 1 year follow-up.

### Intravascular ultrasound grayscale results

**Figure 5:**
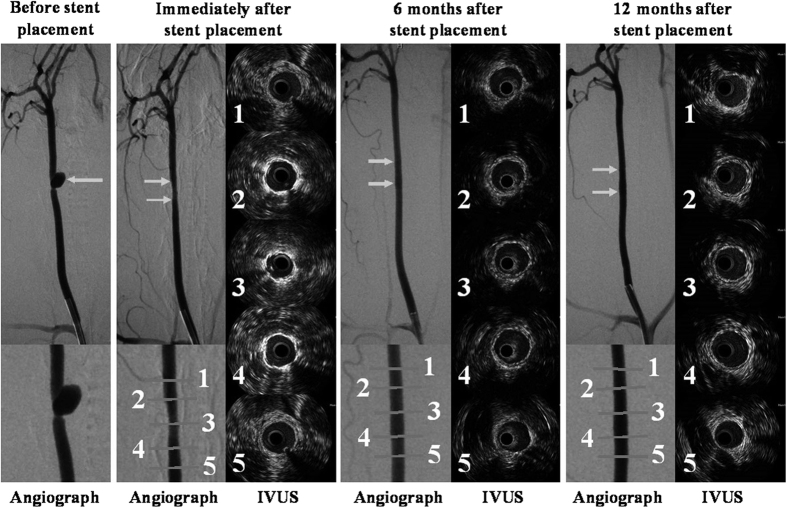
The angiographic and IVUS grayscale results in a lateral aneurysm model in the right CCA managed with a magnesium alloy covered stent. (**A**) Preoperative DSA shows a lateral aneurysm (arrow) at the upper portion of the CCA. (**B**) DSA immediately after stent placement exhibits disappearance of the aneurysm, with slight stenosis of the right CCA (double arrow); (**C**) The IVUS grayscale results of different parts of the normal CCA and stented CCA artery immediately after stent placement. (**D**) DSA 6 months after stent placement shows the disappearance of the aneurysm, without obvious stenosis of the stented CCA. (**E**) The IVUS grayscale results show that the lumen area in the stented CCA is increased compared with the lumen area immediately after stent placement. **(F)** DSA 12 months after stent placement displays disappearance of the aneurysm, with patency of right CCA. (**G**) The IVUS grayscale results also show that the lumen area in the stented CCA has increased as compared with the lumen area immediately after stent placement. Note: 1 = Normal CCA 5 mm distal to the stent; 2 = distal portion of the stented CCA; 3 = middle portion of the stented CCA; 4 = proximal portion of the stented CCA; 1 = normal CCA 5 mm proximal to the stent.

**Figure 6:**
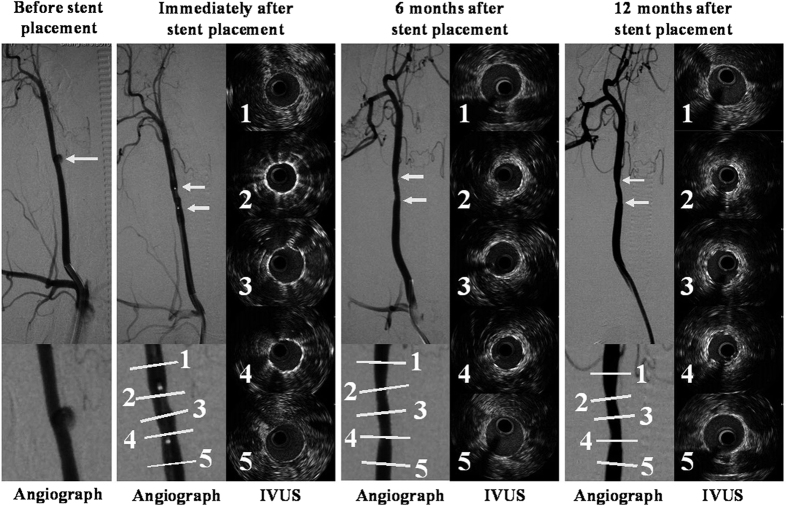
The angiographic and IVUS grayscale results in a lateral aneurysm model in the right CCA managed with a Willis covered stent. (**A**) Preoperative DSA shows a lateral aneurysm (arrow) at the upper portion of the CCA. (**B**) DSA immediately after stent placement exhibits disappearance of the aneurysm, in the right CCA with the balloon (double arrow) still in place in the right CCA. (**C**) The IVUS grayscale results of different parts of the normal CCA and stented CCA immediately after stent placement. (**D**) DSA 6 months after stent placement shows the disappearance of the aneurysm, with slight stenosis of the stent CCA. (**E**) The IVUS grayscale results show that the lumen area in the stented CCA is decreased compared with the lumen area immediately after stent placement. **(F)** DSA 12 months after stent placement displays disappearance of the aneurysm, with stenosis of the stented CCA. (**G**) The IVUS grayscale results also show that the lumen area in the stented CCA is decreased compared with the lumen area immediately after stent placement. Note: 1 = Normal CCA 5 mm distal to the stent; 2 = distal portion of the stented CCA; 3 = middle portion of the stented CCA; 4 = proximal portion of the stented CCA; 1 = CCA 5 mm proximal to the stent.

The IVUS grayscale results after stent placement are presented in Table 3. The IVUS images of the magnesium alloy covered stent and Willis covered stent immediately after stent placement and at 6 and 12 months follow-ups are presented in Figs 5 and 6, respectively.

Compared with the measurements taken immediately after stent placement, the mean vessel area, the mean scaffold area, and the mean lumen area were significant increased at 6 and 12 months follow-ups in group A (*P *< 0.05). However, no significant differences from baseline values were found in the mean lumen stenosis rate in the stented CCAs at 6 and 12 months in group A.

In group B, the mean vessel area of the stented CCAs at 6 and 12 months were significantly greater than that at stent placement (*P *< 0.05), but the mean lumen areas at 6 and 12 months were significant smaller than that at stent placement (*P* < 0.05). Moreover, in group B, compared with the baseline value, the mean lumen stenosis rate was significantly increased at 6 and 12 months.

In addition, although, the mean lumen area in group A was significantly smaller than that in group B immediately after stent placement (*P *< 0.05), it was significantly bigger in group A at 6 and 12 months follow-ups (*P* < 0.05).

## Discussion

This study was designed to compare the *in vivo* efficacy of the two covered stents for treatment of lateral aneurysm in rabbit CCA. The main findings of the study are the following: (1) the magnesium alloy covered stent, with magnesium alloy stent degradation over time, is an effective approach for occlusion of the lateral aneurysm model in rabbit CCA; (2) in group A, in which the magnesium alloy covered stents were used, the mean diameters of the stented CCAs at 6 and 12 months were significantly greater than that at stent placement, moreover, the mean diameter of the stented CCAs in group A was significantly greater than the mean diameter in group B at 6 or 12 months follow-ups. (3) IVUS exhibited significantly greater mean lumen area of the stented CCAs in group A at 6 and 12 months than that immediately stent placement; moreover, the mean lumen area of the stented CCAs in group A at 6 and 12 months was significantly greater than that of group B at 6 or 12 months follow-ups. (4) The lumen stenosis rate in group A immediately after stent placement and at 6 and 12 months follow-ups were almost the same, but in group B it was increased at all three time points.

In this study, placement of the magnesium alloy covered stent was technically successful in all the rabbits, with no procedure-related complications. Complete occlusion of the aneurysms was achieved in all rabbits, without evidence of recanalization or obvious stenosis on the final angiograms. The significant improvement in complete occlusion rates observed during the angiographic follow-up seems to be predominantly attributable to the occlusion of the orifice of the aneurysms. These results indicate that the magnesium alloy covered stent is an effective option for the treatment of a lateral aneurysm in rabbit CCA.

In group A, the significant increase in the mean diameter and mean vessel area of the stented CCAs at 6 and 12 months follow-ups seems to be predominantly attributable to the degradation of the magnesium alloy stent. The mean diameter, mean vessel area, and mean lumen area of the stented CCAs increased significantly after stent placement. The mechanical integrity of the magnesium alloy stent used in this study is only maintained for 30 days (as we have demonstrated with micro-CT in another (unpublished) study); thereafter, as the stent degrades, the radial force exerted by the stent is progressively lost, and the mean diameter and the mean vessel area are free to increase with the growth of the vessel. Although the e-PTFE membrane persists in the vessel, it does not limit the growth of the vessel because it is soft and the diameter (Φ3 mm) of the stent is larger than that of the vessel. In addition, the original volume of the magnesium alloy stent strut decreases with its degradation, which also contributes to the increase of the mean lumen area to a certain extent. This phenomenon of increase in lumen area with degradation of the magnesium alloy stent is consistent with the results of the DREAMS study^15^.

Unlike in group B in which a significant decrease in mean lumen area was seen at 6 and 12 months follow-ups, the mean lumen area in group A at 6 and 12 months follow-ups was greater than that immediately after stent placement. One reason for the difference between the two stents in the mean lumen area may be related to the tissue reaction associated with the stents. The magnesium alloy stent tends to prevent platelet accumulation^8^,^16^, which would result in less intimal hyperplasia than is seen with the Willis covered stent. Another reason may be the degradation of the magnesium alloy stent. As the stent degrades, the radial force decreases, allowing the vessel in the stented area to grow along with the growth of the CCAs. On the other hand, the persistent stimulation and vessel limitation due to the stent, as well as the absence of an anti-platelet aggregation effect, of the Willis covered stent results in decrease in the lumen area until the completion of endothelialization.

The unique characteristic of this study was the evaluation of the degradation of the magnesium alloy stent *in vivo*. First, we checked the degradation of the Nd on the surface of the stent by MRA, which occurred over 4–5 weeks. Second, we observed the degradation of the magnesium alloy stent over time with molybdenum target examination. Disintegration and outline ambiguity of the stent with time was observed during follow-up. Although we did not observe the complete degradation of the stent, we have described an approach that can be used for the monitoring of stent degradation *in vivo*.

This study has several limitations. First, the number of lateral aneurysms treated was small. Bigger trials are required to determine the long-term outcomes. Second, due to the thin caliber of the CCAs in the rabbit, the neck of the lateral aneurysms was narrow and easily obliterated. Whether a wide-necked aneurysm or an aneurysm in a curved artery could be occluded with the magnesium alloy covered stent needs further studies. Third, we focused on *in vivo* findings in this article and did not compare the results of IVUS with histomorphometry, mainly because differences in *ex vivo* and *in vivo* measurements are possible due to shrinkage of the vessel during postmortem processing of the stented segments, especially in the case of an artery in which a magnesium alloy covered stent has undergone degradation. Last, the magnesium alloy stent in the present study was naked, without any coating, and so its mechanical integrity was only partly maintained after 30 days, which did not meet the clinical requirement of 3–4 month vessel supporting time. Further research is necessary to examine whether the use of an appropriate biodegradable coating on the surface of the magnesium stent can protect the stent substrate from rapid degradation.

In conclusion, the magnesium alloy covered stent, with degradation of the magnesium alloy stent over time, proved to be an effective approach for occlusion of a lateral aneurysm in the rabbit CCA. The magnesium alloy covered stent provides distinct advantages with regard to the growth of the diameter and lumen area of the stented artery, with results that are comparable to those obtained with the Willis covered stent.

## Additional Information

**How to cite this article**: Wu, W. *et al*. Magnesium alloy covered stent for treatment of a lateral aneurysm model in rabbit common carotid artery: An in vivo study. *Sci. Rep.* **6**, 37401; doi: 10.1038/srep37401 (2016).

**Publisher’s note:** Springer Nature remains neutral with regard to jurisdictional claims in published maps and institutional affiliations.

## Figures and Tables

**Table 1:** The composition of the magnesium alloy.

Element	Mg	Nd	Zn	Zr	Mn	Si	Cu	Fe
wt.%	Balance	2.1	0.21	0.5	0.009	0.006	0.005	0.002

**Table 2:** Angiographic findings after stent placement.

Group	No. of Animals	Location	Diameter of the stented CCA (mm)		
			Immediately	6 months	12 months
A	7	Distal	2.40 ± 0.03	2.56 ± 0.13*^†^	2.58 ± 0.13*^†^
	7	Middle	2.31 ± 0.14	2.63 ± 0.10*^†^	2.65 ± 0.07*^†^
	7	Proximal	2.41 ± 0.09	2.69 ± 0.06*^†^	2.70 ± 0.08*^†^
	7	Average	2.37 ± 0.06	2.63 ± 0.07*^†^	2.64 ± 0.06*^†^
B	5	Distal	2.38 ± 0.05	2.26 ± 0.06*	2.16 ± 0.08*
	5	Middle	2.44 ± 0.12	2.33 ± 0.07*	2.29 ± 0.06*
	5	Proximal	2.43 ± 0.05	2.29 ± 0.05*	2.24 ± 0.05*
	5	Average	2.42 ± 0.03	2.29 ± 0.04*	2.23 ± 0.07*

^*^Diameter of the stented CCAs at 6 and 12 months compared with that immediately after stent placement in the same stented locations (P < 0.05).

^†^Comparison of the same stented sections between group A and group B (*P *< 0.05).

**Table 3:** IVUS graysacle results of the stented sections of the two groups after stent placement.

Group		Vessel area (mm^2^)	Scaffold area (mm^2^)	Lumen area (mm^2^)	Lumen stenosis (%)
A	Immediately	8.38 ± 0.19	6.55 ± 0.10	4.46 ± 0.28†	21.59 ± 3.40
	6 months	10.66 ± 0.08*	8.07 ± 0.54†	5.45 ± 0.24*^†^	23.12 ± 1.90
	12 months	11.00 ± 0.17*	8.30 ± 0.46†	5.73 ± 0.26*^†^	28.49 ± 1.27*
B	Immediately	7.68 ± 0.23	6.49 ± 0.19	5.15 ± 0.25	15.72 ± 1.89
	6 months	10.93 ± 0.11*	6.49 ± 0.21	4.69 ± 0.19	22.40 ± 0.79*
	12 months	11.22 ± 0.30*	6.52 ± 0.19	4.43 ± 0.27*	26.60 ± 2.46*

^*^Comparison of the vessel area and the lumen area of the stented CCAs at 6 and/or 12 months with that immediately after stent placement (*P* < 0.05) CCAs.

^†^Comparison of the vessel area and the lumen area of the stented at the same times between group A and group B (*P* < 0.05).
